# Histone deacetylase 3 overexpression in human cholangiocarcinoma and promotion of cell growth via apoptosis inhibition

**DOI:** 10.1038/cddis.2016.457

**Published:** 2017-06-01

**Authors:** Yuyao Yin, Mingming Zhang, Robert Gregory Dorfman, Yang Li, Zhenguo Zhao, Yida Pan, Qian Zhou, Shan Huang, Shimin Zhao, Yuling Yao, Xiaoping Zou

**Affiliations:** 1 Department of Gastroenterology, Nanjing Drum Tower Hospital, The Affiliated Hospital of Nanjing University Medical School, Nanjing University, Nanjing, China; 2 Northwestern University Feinberg School of Medicine, Chicago, IL, USA; 3 Department of Surgery, The Affiliated Jiangyin Hospital of Southeast University Medical College, Jiangsu, China; 4 Department of Digestive Diseases of Huashan Hospital, Fudan University, Shanghai, China; 5 School of Life Sciences, Fudan University, Shanghai, China; 6 Department of Pathology, The Second Hospital of Anhui Medical University, Anhui Medical University, Hefei, China

## Abstract

Histone deacetylase 3 (HDAC3) has an oncogenic role in apoptosis and contributes to the proliferation of cancer cells. MI192 is a novel HDAC3-specific inhibitor that displays antitumor activity in many cancer cell lines. However, the role of HDAC3 and the antitumor activity of its inhibitor MI192 are not known in cholangiocarcinoma (CCA). The present study aims to identify the target of MI192 in CCA as well as evaluate its therapeutic efficacy. CCK8 and colony formation assays showed that HDAC3 overexpression promotes proliferation in CCA cell lines. HDAC3 knockdown or treatment with MI192 decreased CCA cell growth and increased caspase-dependent apoptosis, while apoptosis was partially rescued by HDAC3 overexpression. We demonstrated that MI192 can inhibit the deacetylation activity of HDAC3 and its downstream targets *in vitro*, and MI192 inhibited xenograft tumor growth *in vivo.* Immunochemistry showed that HDAC3 was upregulated in CCA tissues compared with adjacent normal tissues, and this was correlated with reduced patient survival. Taken together, these results demonstrate for the first time that MI192 targets HDAC3 and induces apoptosis in human CCA cells. MI192 therefore shows the potential as a new drug candidate for CCA therapy.

## Introduction

Cholangiocarcinoma (CCA) is a highly malignant adenocarcinoma with increasing mortality in many countries.^1, 2, 3^ Most patients are diagnosed at late stages and are not eligible for surgical resection or liver transplantation. As a result, 5-year survival rates of CCA remain at 10% for the past three decades.^4^ In addition, as resistance to conventional chemotherapy is becoming increasingly commonplace, the research aimed at developing new strategies for treating CCA in the clinic, as well as identifying new tumor markers, is urgently needed.

Histone acetylation is typically associated with increased transcription, and histone deacetylases (HDACs) are regulatory enzymes that catalyze the removal of acetyl groups from histones. According to their homology, the 11 HDACs can be divided into Classes I, II and IV.^5^ Class I HDACs (1, 2, 3 and 8) have an important role in tumorigenesis and may be candidate targets for many cancer treatments.^6, 7, 8, 9, 10^ Numerous reports indicate that Class I HDACs are overexpressed in many cancers and inhibit specific tumor suppressor genes, resulting in an aberrant epigenetic status compared with adjacent normal cells.^11, 12^ Recently, studies showed that high levels of HDAC3 expression and activity had a critical role in cell epigenetic alterations associated with malignancies.^13, 14^ However, the role of HDAC3 in CCA has not been elucidated.

HDAC inhibitors show great potential as promising chemotherapeutic agents because acetylation-mediated epigenetic changes are reversible. Indeed, numerous HDAC inhibitors (Vorinostat (Suberoylanilide Hydroxamic Acid; SAHA), AR-42, romidepsin, entinostat and valproic acid) exhibit antitumor effects in a variety of tumors both *in vitro* and *in vivo*.^15, 16, 17, 18^ Two HDAC inhibitors, SAHA and romidepsin, are US Food and Drug Administration (FDA) approved for the treatment of cutaneous T-cell lymphoma.^19^ MI192, a novel HDAC3-selective inhibitor, was found to be beneficial for rheumatoid arthritis *in vitro* with marginal toxicity.^20^ However, the effects of MI192 in CCA have not yet been studied.

Here we confirmed high levels of HDAC3 in CCA tissues, suggesting poor survival in patients with CCA. We also found that high levels of HDAC3 induced proliferation as well as inhibited apoptosis in CCA cell lines. MI192 inhibited cancer cell proliferation through the induction of cell apoptosis primarily through targeting HDAC3. Thus, HDAC3 inhibition caused apoptosis in CCA cells, and selective inhibition of HDAC3 through novel inhibitors may be useful for CCA therapy.

## Results

### HDAC3 promoted growth in CCA cells

**Figure 1:**
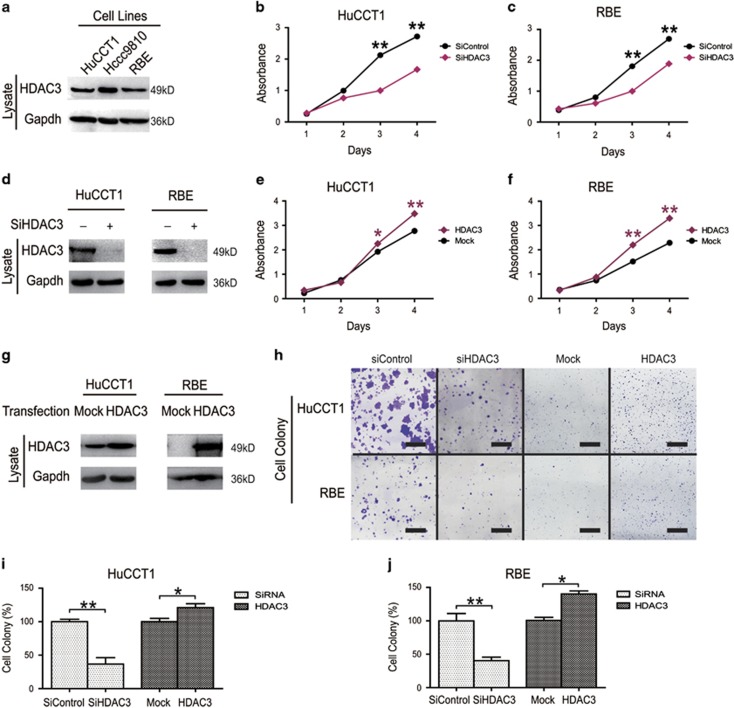
HDAC3 promoted growth in CCA cells. (**a**) HDAC3 protein levels were detected by western blot. (**b** and **c**) HuCCT1 and RBE cell proliferation was analyzed via CCK8 assay following small interfering RNA (siRNA) transfection. (**d**) Transfection efficiency was confirmed by western blotting. (**e** and **f**) HuCCT1 and RBE cell proliferation was analyzed via CCK8 assay following transfection with HDAC3 overexpression vector. (**g**) Transfection efficiency was confirmed by western blotting. (**h**) Cells were transfected with HDAC3 overexpression vector or siRNAs, and then colonies were stained with crystal violet and photographed. Scale bars, 1 cm. (**i** and **j**) Stained colonies were quantified. Data represent the mean±S.E.M., *n*≥3. *P<0.05 and **P<0.01

Recently, studies showed that high levels of HDAC3 expression and activity had a critical role in cell epigenetic alterations associated with malignancies.^13, 14^ However, the role of HDAC3 in CCA has not been elucidated. We assessed the expression of HDAC3 in CCA cell lines, and found HDAC3 significantly expressed in all three CCA cell lines (Figure 1a). Next, we evaluated the relationship between cell proliferation and HDAC3 expression using two human CCA cell lines (HuCCT1 and RBE). Transfection of cells with siHDAC3 significantly decreased CCA cell proliferation (Supplementary Figures 1A and Figures 1c–e), whereas HDAC3 overexpression increased CCA cell proliferation (Figures 1g–h). Consistent with proliferation studies, transfection of cells with siHDAC3 significantly decreased colony formation, and HDAC3 overexpression significantly increased CCA cell clonogenicity (Figures 1i–k). Collectively, these results indicate that HDAC3 had a key role in promoting cell proliferation.

### The inhibitory effect of MI192 on CCA cell viability

**Figure 2:**
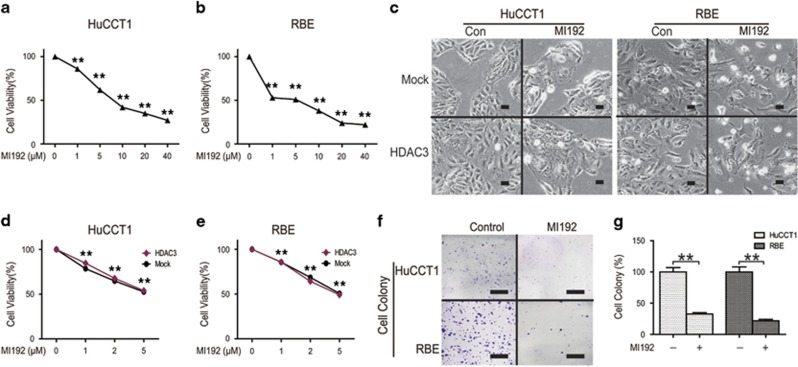
The inhibitory effect of MI192 on CCA cell viability. (**a** and **b**) Cells were treated with MI192 and quantified via CCK8 assay. (**c**) Cells were treated with MI192 and morphological changes were observed. The magnification is x200. Scale bars, 100 *μ*m. (**d** and **e**) Cells were transfected with HDAC3 overexpression vector, and then treated with MI192 and quantified via CCK8 assay. (**f** and **g**) Cells were treated with MI192. Colonies were stained with crystal violet (left) and quantified (right). Scale bars, 1 cm; **P<0.01

CCK8 assay results showed that the 50% growth inhibitory concentration (IC_50_) of MI192 at 48 h was ~6 *μ*M in HuCCT1 cells and RBE cells (Figures 2a–c). Although transfection of cells with HDAC3 plasmid significantly increased cell growth, HDAC3 overexpression hardly reversed the inhibitory effect of MI192 on CCA cells (Figures 2d and e). Consistent with CCK8 assays, colony formation in CCA cells was significantly decreased after MI192 treatment (Figures 2f and g). Collectively, these results reveal that MI192 treatment reduced CCA cell viability.

### MI192-induced CCA cell apoptosis *in vitro*

**Figure 3:**
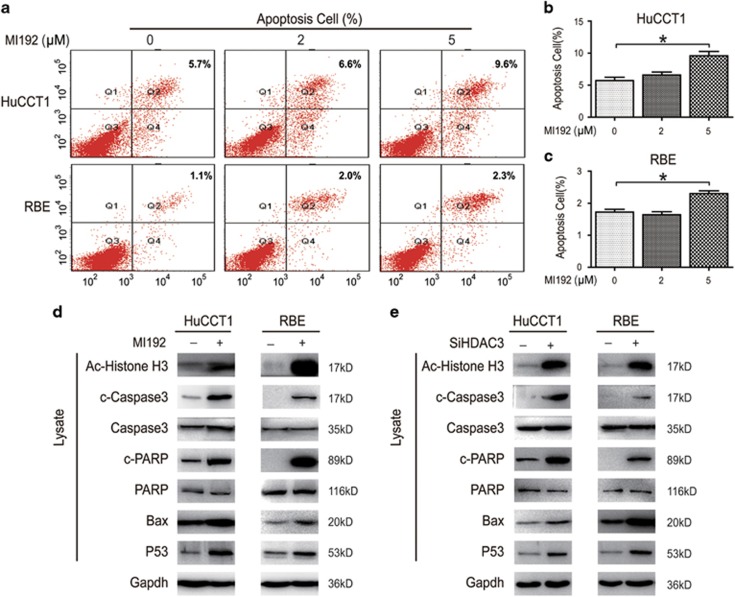
MI192-induced CCA cell apoptosis *in vitro*. (**a**) Cells were treated with MI192, and cell apoptosis was analyzed via flow cytometry. (**b** and **c**) Cell apoptosis was quantified. (**d** and **e**) Cells were collected and subjected to western blot after MI192 treatment (left) and small interfering RNA (siRNA) transfection (right); *P<0.05

To further explore the mechanism of MI192-induced cell proliferative inhibition, flow cytometry was used, and results demonstrated that MI192 increased the relative amount of cell apoptosis (Figures 3a–c). We investigated the effects of HDAC3 on their downstream targets following MI192 treatment, including acetylated *α*-histone3, P53 and Bax.^21^ MI192-induced caspase substrate (polyADP ribose polymerase (PARP) and caspase-3) cleavage and P53 expression were mimicked by HDAC3 knockdown (Figures 3d and e). Altogether, these results indicate that HDAC3 participated in MI192-induced apoptosis in CCA cells and that HDAC3 might be the target of MI192.

### HDAC3 is the direct target of MI192

**Figure 4:**
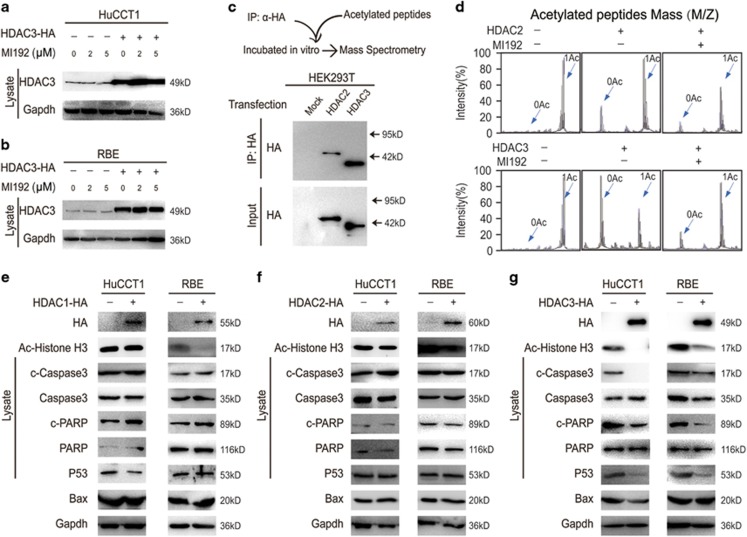
HDAC3 is the direct target of MI192. (**a** and **b**) HDAC3-overexpressing cells were treated with MI192 and subjected to western blot. (**c**) Schematic diagram of the *in vitro* deacetylation assay with HDAC3 (top). The immunoprecipitated protein corresponding to HDAC-HA was subjected to western blot (bottom). (**d**) The HDAC protein was incubated with acetylated peptides with or without MI192, and the rate of deacetylation was determined using mass spectrometry (MS). (**e**) HDAC1-overexpressing cells and their counterparts were subjected to western blot. (**f**) HDAC2-overexpressing cells and their counterparts were subjected to western blot. (**g**) HDAC3-overexpressing cells and their counterparts were subjected to western blot. Data represent the mean±S.E.M., *n*≥3. *P<0.05 and **P<0.01; NS not significant

To determine whether MI192 inhibited HDAC3 activity, CCA cells were treated with MI192 following transfection with a HA-tagged HDAC3 vector. We found that HDAC3 protein levels were unchanged by MI192 treatment (Figure 4a). Inhibition of HDAC3 activity by MI192 was subsequently assessed by evaluating specific acetylation of histone H3, the downstream target of HDAC3.^21^ We found that MI192 increased specific histone H3 acetylation, and HDAC3 overexpression reversed this effect (Figures 4a, b and d).

Because of the high level of homology between the Class I HDACs, HDAC2 shares 52% identity with HDAC3,^22, 23, 24^ and MI192 possibly has a weak inhibitory effect on HDAC2.^20, 25^ We then attempted to determine whether HDAC3 was responsible for MI192-induced apoptosis. To elucidate the direct target of MI192, we used an *in vitro* deacetylation system (Figure 4c). MI192 treatment inhibited HDAC3 deacetylation activity, but only had a marginal inhibitory effect on HDAC2 (Figure 4d). We investigated the effects of HDACs 1, 2 and 3 on apoptosis-related targets and found that only HDAC3 could rescue the apoptosis signal in CCA cell lines (Figures 4e–g). These data suggest that MI192 inhibited the HDAC3 activity.

### Effects of MI192 on tumor xenografts

**Figure 5:**
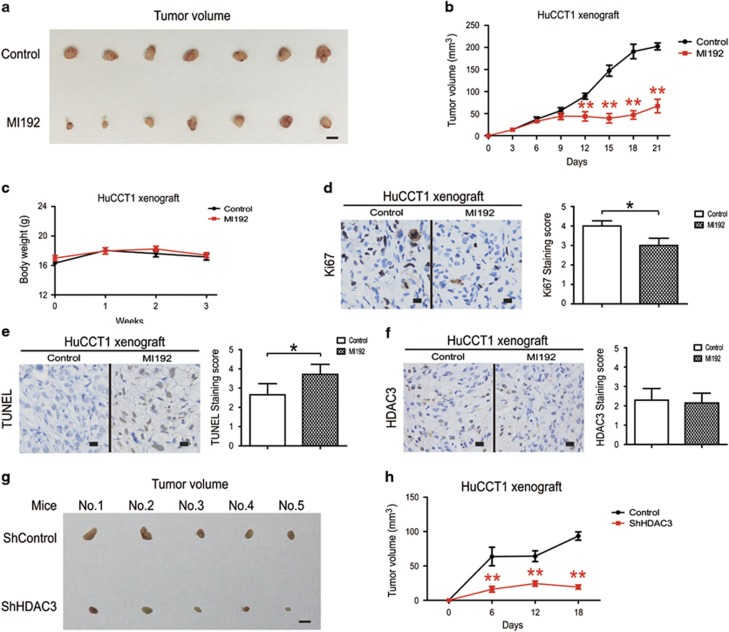
Effects of MI192 on tumor xenografts. (**a**) Systemic delivery of MI192 suppresses CCA cell xenograft tumor growth in nude mice. Tumors were photographed after all animals were killed. Scale bars, 1 cm. (**b**) The xenograft tumor sizes. (**c**) The body weights of tumor-burdened mice. (**d**) Xenograft samples were stained with Ki-67 (left) and staining was quantified (right). The magnification is x200. Scale bars, 100 *μ*m. (**e**) Xenograft samples were stained with TUNEL (left) and staining was quantified (right). The magnification is x200. Scale bars, 100 *μ*m. (**f**) Xenograft samples were stained with HDAC3 (left) and staining was quantified (right). The magnification is x200. Scale bars, 100 *μ*m. (**g**) HDAC3 knockdown HuCCT1 cells and control counterparts were injected at the left and right sides of the same mice. Tumors were photographed after all animals were killed. Scale bars, 1 cm. (**h**) Xenograft tumor sizes. Data represent the mean±S.E.M., *n*≥3. *P<0.05 and **P<0.01; NS not significant

We used a CCA cell tumor xenograft model to evaluate the *in vivo* anticancer and HDAC3 inhibitory activity of MI192, and found that MI192 administration significantly inhibited tumor growth (Figures 5a and b). The body weights of treated mice were used as indicators of health.^26^ MI192 treatment did not affect mouse body weight, suggesting that the mice did not experience evident toxicity *in vivo* (Figure 5c). Furthermore, histological sections of xenograft samples were stained with TUNEL, c-caspase-3 and Ki-67, markers of cell apoptosis and proliferation, respectively.^26^ Consistent with the *in vitro* results, MI192 administration increased TUNEL and c-caspase-3 staining and reduced Ki-67 staining in xenograft tissues, confirming the antitumor effect of MI192 (Supplementary Figures 1E, Figures 5d and e).

Although other Class I HDACs are found primarily in the nucleus, HDAC3 can shuttle in and out of the nucleus as its catalytic domain is positioned much closer to the C terminus than other Class I HDACs.^23^ We found that HDAC3 mainly localized to the nucleus, but was also observed in the membrane. MI192 treatment did not significantly change the location and protein level of HDAC3 in CCA cell xenograft samples (Figure 5f). Consistent with previous results, we also found that xenografts from HDAC3 knockdown cells were significantly smaller than their counterparts (Supplementary Figures 1C, D, Figures 5g and h). Altogether, these data demonstrate the apoptosis-inducing and proliferation-inhibiting activity of MI192 *in vivo*.

### HDAC3 expression was increased in CCA tissues and associated with reduced patient survival

**Figure 6:**
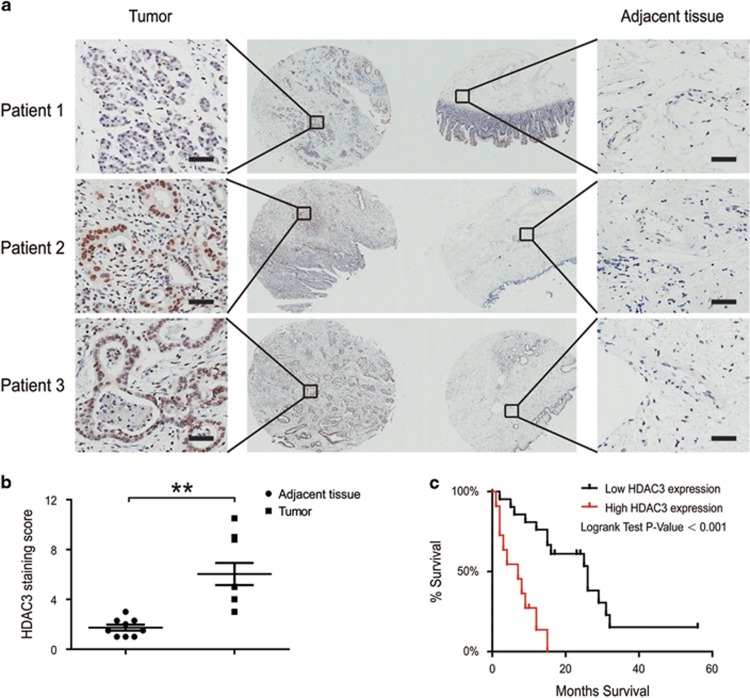
HDAC3 expression was increased in CCA tissues. (**a** and **b**) HDAC3 protein levels in tumor and adjacent normal tissues from nine CCA patients were detected (top) and quantified (bottom). The magnification is x200. Scale bars, 100 *μ*m. (**c**) The 5-year survival was reduced for CCA patients with elevated HDAC3 protein expression. Data represent the mean±S.E.M., *n*≥3. *P<0.05 and **P<0.01

Class I HDACs (especially 1, 2 and 3) have an important role in tumorigenesis and numerous reports indicate that Class I HDACs are overexpressed in many cancers, resulting in an aberrant epigenetic status compared with adjacent normal cells.^11, 12^ We queried the tissue microarrays from Shanghai Outdo Biotech (Shanghai, China), which contains clinically annotated data from 127 CCA samples. When we assessed the expression of HDAC3 on nine pairs of CCA tissues, we found that HDAC3 significantly promoted in tumor tissues compared with adjacent tissues (Figures 6a and b). We evaluated the tissue microarrays, which contain clinically annotated genomic data from CCA samples, and found that HDAC3 protein was overexpressed in 47/127 CCA cases (37%), and was associated with tumor size (Table 1). Using the 33 follow-up cases, we found that high HDAC3 protein in CCA reduced patient survival (*P*<0.001, log-rank test) (Figure 6c).

## Discussion

Numerous reports indicate that HDACs are overexpressed in many cancers and inhibit specific tumor suppressor genes, thereby resulting in aberrant epigenetics in cancer cells.^11, 12^ Among them, Class I HDACs have important roles in tumorigenesis. This makes Class I HDACs promising targets for antitumor therapeutics.^6, 7, 8, 9, 10^ As modifications in HDAC8 expression did not affect cancer cell proliferation^27, 28^ and expression of Class I HDACs in CCA has not yet been studied, we set out to determine the expression of Class I HDACs (especially HDACs 1, 2 and 3) in CCA tissue.

Using immunohistochemistry, we found that the expression of HDAC3 was differentially expressed and correlated with clinicopathological factors in CCA. We also screened for the effects of HDAC3 on CCA cell proliferation and confirmed that HDAC3 enhances cell proliferation as well as inhibits apoptosis, indicating that HDAC3 could be a potential target of the chemotherapeutic HDAC3 inhibitor.

HDACs 1 and 2 share 82% identity with each other, as well as share 53% and 52% identity with HDAC3, respectively.^22, 23, 24^ Owing to the high level of homology between the Class I HDACs, it is easy to understand why an HDAC3-selective inhibitor would be difficult to identify. Although MI192, a new class of inhibitor, can show higher selectivity for HDAC3 over HDACs 1 and 2,^20, 29^ its inhibitory effects on other HDACs besides HDAC3 could not be ignored. Therefore, we evaluated the inhibitory effect of MI192 on HDACs 2 and 3 by using mass spectrometry, and confirmed that MI192 could only significantly inhibit HDAC3 *in vitro*. At the molecular level, HDAC3 overexpression not only partially reversed cell apoptosis but also reversed apoptosis-related proteins, whereas HDAC1 and 2 did not show a similar effect. Consistent with *in vitro* data, MI192 significantly inhibited the *in vivo* activity of HDAC3 and induced apoptosis in Hucct1 xenograft tissues. These data suggest that MI192 induces CCA cell apoptosis by inhibiting the activity of HDAC3.

As the catalytic domain of HDAC3 is positioned much closer to the C terminus than other Class I HDACs, the structure of HDAC3 is distinct from other Class I HDACs.^23^ This may explain why HDAC3 protein can shuttle in and out of the nucleus, whereas other Class I HDACs are found primarily in the nucleus.^23^ Studies have shown that phosphorylation of a specific serine residue in the HDAC3 protein is regulated by c-Src, kinase CK2 and phosphatase PP4, and that phosphorylation contributes to HDAC3 activity as well as relocation.^30, 31^ To elucidate the impact of MI192 on HDAC3, we found that MI192 treatment did not significantly change the cellular location or protein level of HDAC3 in CCA cells and xenograft samples, indicating that MI192 inhibits the deacetylation activity of HDAC3 as opposed to its expression and phosphorylation.

Acetylation increases p53 protein stability, and upon acetylation of p53 at K120, p53 preferentially activates the expression of proapoptotic genes *BAX*, *PUMA*, *DR5* and *NOXA*.^32^ We evaluated the role of P53 in HDAC3-related apoptosis and found that both HDAC3 knockdown and MI192 treatment significantly increased protein levels of P53 and activated the expression of the downstream proapoptotic gene *BAX*. HDAC3 overexpression not only rescued cell apoptosis but also reversed the upregulation of p53 and BAX in CCA cells, indicating that MI192 promotes CCA cell apoptosis partially by increasing HDAC3 acetylation of p53.

In conclusion, the present work found that HDAC3 is a key regulatory factor for cancer proliferation and apoptosis, and is associated with poor prognosis in CCA patients. MI192, as an HDAC inhibitor, represents a novel treatment approach for CCA, and isoform-selective HDAC3 inhibition may improve therapeutic margins of safety. Further characterization of HDAC inhibitors is needed to better establish their role in the management of CCA.

## Materials and methods

### Ethics, consent and permissions

All experiments using animal and human samples were approved by the Ethical Committee of Medical Research, Nanjing Drum Tower Hospital, Affiliated Hospital of Nanjing University Medical School.

### Cell culture and reagents

Three human CCA cell lines were used: HuCCT1, Hccc9810 and RBE. HuCCT1 and Hccc9810 were obtained from the Japanese Collection of Research Bioresources (JCRB) (Tokyo, Japan). RBE was obtained from the Institute of Biochemistry and Cell Biology, Shanghai Institutes for Biological Sciences, Chinese Academy of Sciences (Shanghai, China). Cells were maintained in RPMI-1640 (Invitrogen, Carlsbad, CA, USA) containing 10% fetal bovine serum (Invitrogen), penicillin (Invitrogen) (100 U/ml) and streptomycin (Invitrogen) (100 U/ml). MI192 (Sigma, St. Louis, MO, USA) was commercially purchased.

### Immunohistochemistry

Tumor specimens were fixed in 4% formalin and embedded in paraffin. The sections were incubated with TUNEL Kit Buffer (Gugebio, Wuhan, China), anti-active-caspase-3 (Abcam, Cambridge, UK) or anti-Ki-67 antibodies (Santa Cruz, Dallas, TX, USA), and subsequently with DAPI (Gugebio) as well as the corresponding secondary antibody (Zsbio, Beijing, China). Sections were treated with immunoperoxidase using the DAB Kit (Zsbio) and then scored.^33^ The tissue microarray slides were obtained from Shanghai Outdo Biotech. Staining intensity was graded as follows: absent staining=0, weak=1, moderate=2 and strong=3. The percentage of staining was graded as follows: 0 (no positive cells), 1 (<25% positive cells), 2 (25–50% positive cells), 3 (50–75% positive cells) and 4 (>75% positive cells). The score for each tissue was calculated by multiplying, and the range of this calculation was therefore 0–12.^34^

### Cell transfection

Cells were transfected using Lipofectamine 3000 (Invitrogen) according to the manufacturer’s protocol. The HDAC3 siRNAs were commercially purchased from RiboBio (Guangzhou, China), siRNA-HDAC3-1: 5′-CCATGACAATGACAAGGAA-3′, siRNA-HDAC3-2: 5′-GCATTGATGACCAGAGTTA-3′, siRNA-HDAC3-3: 5′-GAATATGTCAAGAGCTTCA-3′. HDAC3 shRNA (h) lentiviral particles were commercially purchased (Santa Cruz). The control vector, HDAC1–3 expression vectors were kindly provided by the Zhao lab of Fudan University (Shanghai, China).

### Western blotting analysis

Cells were lysed with 0.5% NP-40 lysis buffer and proteins were blotted following the standard protocol. Signals were probed using the Chemiluminescence ECL Plus Reagent (Thermo, Grand Island, NY, USA), as well as detected using the Chemiluminescence HRP Substrate (Millipore, Billerica, MA, USA) and Tanon 5200Multi Scanner (Shanghai, China). Primary antibodies were as follows: HDAC1 (Abcam), HDAC2 (Abcam), HDAC3 (Abcam), cleaved caspase-3 (CST, Danvers, MA, USA), cleaved PARP (CST), PARP (CST), GAPDH (Bioworld, St. Louis Park, MN, USA), K9 acetyl-histone H3 (CST), Bax (CST), P53 (Santa Cruz), PUMA (CST) and HA (CST).

### Cell viability and clonogenic assay

Cells viability was determined using the CCK8 colorimetric assay in 96-well plates (2 × 10^3^ cells per well) (Dijindo, Minato-ku, Tokyo, Japan). The absorbance at 450 nm was recorded using a microplate reader. For the clonogenic assay, cells were seeded into 6-well plates (5 × 10^2^ cells per well) and cultured for 10 days. Colonies were fixed with 4% paraformaldehyde, stained with crystal violet and then counted.

### Apoptosis assay

Cell apoptosis was measured by flow cytometry using the AnnexinV-FITC/PI Apoptosis Detection Kit (BD, Franklin Lakes, NJ, USA) following the manufacturer's instructions.

### HDAC deacetylation assay

Cells were lysed in NP-40 buffer containing 50 mM Tris-HCl (pH 7.5) (Sigma), 150 mM NaCl (Sangon, Shanghai, China), 0.5% Nonidet P-40 (Sigma), 1 *μ*g/ml aprotinin (Sigma), 1 *μ*g/ml leupeptin (Sigma), 1 *μ*g/ml pepstatin (Sigma), 1 mM Na_3_VO_4_ (Sigma) and 1 mM PMSF (Sigma). For immunoprecipitation, 500 *μ*l of cell lysate was incubated with HA antibody (provided by the Zhao Lab of Fudan University) for 3 h at 4 °C with rotation. Then, 30 *μ*l Protein A Agarose (Millipore) was added for 12 h at 4 °C with rotation, and the beads were washed three times with lysis buffer before proteins were dissolved in loading buffer. Deacetylation assays were carried out in the presence of 5 *μ*g enzyme and 0.3 *μ*g peptide in 30 *μ*l reaction buffer (30 mM HEPES (Sigma), 0.6 mM MgCl_2_ (Sangon), 1 mM DTT (Sigma), 1 mM NAD^+^ (Sigma), 10 mM PMSF (Sigma)). The deacetylation reaction was incubated for 3–5 h at 37 °C before the mixture was desalted by passing it through a C18 ZipTip (Millipore). The desalted samples were analyzed using a MALDI-TOF/TOF mass spectrometer (Applied Biosystems, Grand Island, NY, USA). The acetylated peptide used in the assay was NLASVEELK^Ac^EIDVEVRK (Glssale, Shanghai, China).

### CCA cancer xenograft model

Nude mice were purchased from the Department of Laboratory Animal Science, Nanjing Drum Tower Hospital (Jiangsu Sheng, China). HuCCT1 cells (5 × 10^6^) in FBS-free RPMI-1640 were subcutaneously injected into the flanks of mice. HDAC3 knockdown cells and control counterparts were injected at the left and right sides of the same mice. Once xenograft tumors were palpable, mice were treated with MI192 at a dose of 25 mg/kg body weight in 200 *μ*l volume via intraperitoneal injection twice a week for 3 weeks. Tumor volume was calculated using the formula, length (*L*) x width (*W*) x height (*H*) x 0.5236. The Animal Welfare Committee of Nanjing Drum Tower Hospital approved all procedures involving animals.

### Statistics

Data were expressed as mean±S.E. The data were analyzed through one-way ANOVA followed by *post hoc* Duncan tests (SPSS 17.0, SPSS Inc., Chicago, IL, USA). *P*<0.05 was considered significant.

## Figures and Tables

**Table 1:** Clinical characteristics and HDAC3 levels in patients with cholangiocarcinoma

	***n***	**HDAC3 expression, *n* (%)**		**X^2^**	***P*-value**
		**Low (%)**	**High (%)**		
*Gender*				0.53	0.817
Male	80	51 (63.7)	29 (36.3)		
Female	47	29 (61.7)	18 (38.3)		
					
*Age (years)*				0.045	0.833
≤60	61	39 (63.9)	22 (36.1)		
>60	66	41 (62.1)	25 (37.9)		
					
*Location*				3.216	0.073
Intra	100	59 (59)	41 (41)		
Extra	27	21 (77.8)	6 (22.2)		
					
*Size (mm)*				21.049	<0.001*
≤60	65	53 (81.5)	12 (18.5)		
>60	52	21 (40.4)	31 (59.6)		
					
*Differentiation*				0.041	0.839
Well	107	67 (62.6)	40 (37.4)		
Poor	20	13 (65)	7 (35)		
					
*T stage*				0.057	1
T1–T2	47	32 (68.1)	15 (31.9)		
T3	14	10 (71.4)	4 (28.6)		
					
*Lymph node metastasis*				0.168	0.682
Negative	48	33 (68.8)	15 (31.3)		
Positive	25	16 (64)	9 (36)		
					
*Venous invasion*				0.065	0.798
Negative	112	71 (63.4)	41 (36.6)		
Positive	15	9 (60)	6 (40)		
					
*Nerve invasion*				5.709	0.032*
Negative	115	69 (60)	46 (40)		
Positive	12	11 (91.7)	1 (8.3)		

Abbreviation: HDAC3, histone deacetylase 3.
