# Supplementary figures and images for: Histone deacetylase 3 overexpression in human cholangiocarcinoma and promotion of cell growth via apoptosis inhibition
Source: Cell Death Dis. 2017 Jun 1;8(6):e2856–. doi: 10.1038/cddis.2016.457 (PMC5520875; doi:10.1038/cddis.2016.457)

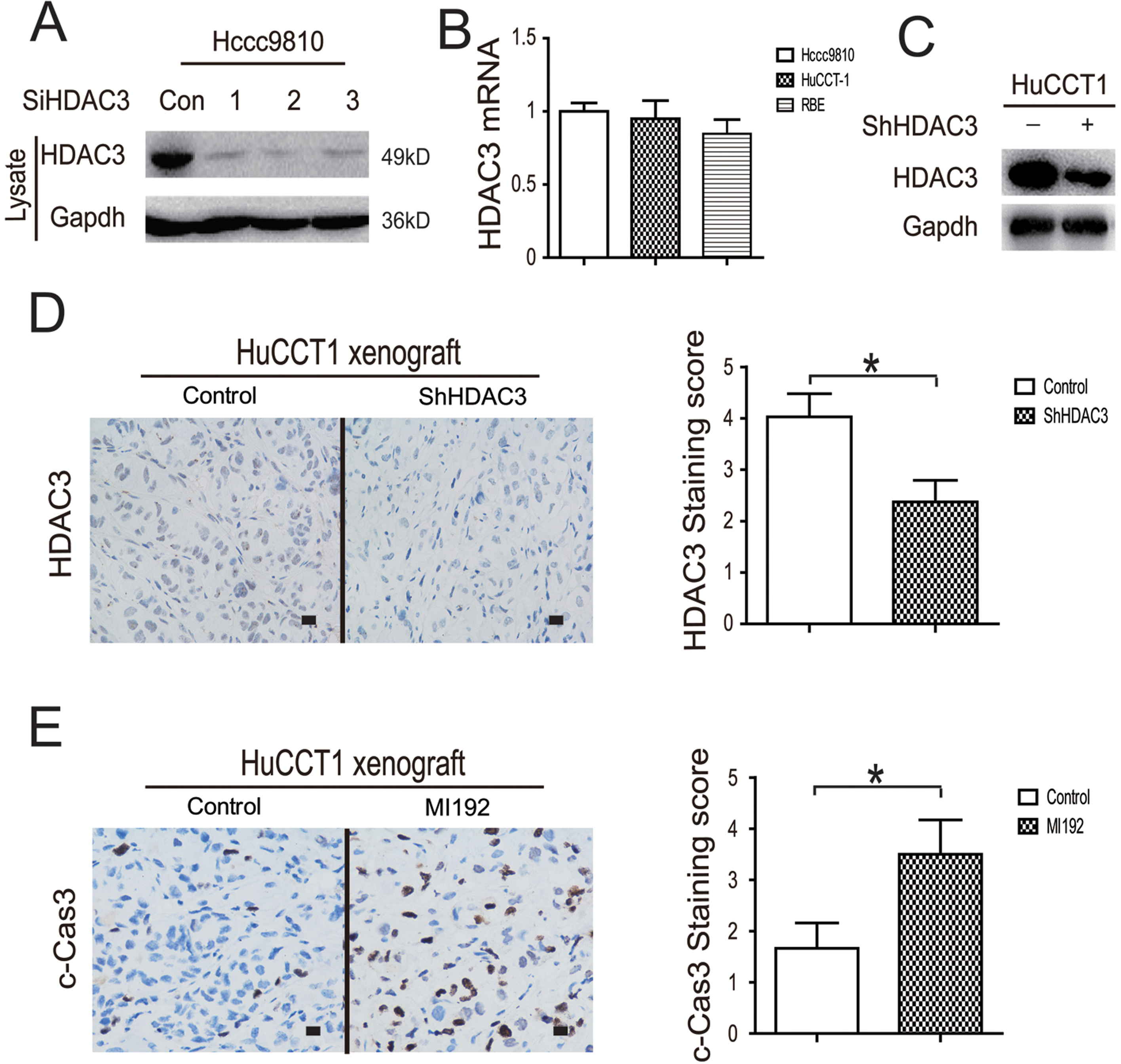

Supplement: Supplementary Figure [file cddis2016457x1.tif]
