# Supplementary material for: Histone deacetylase 3 overexpression in human cholangiocarcinoma and promotion of cell growth via apoptosis inhibition
Source: Cell Death Dis. 2017 Jun 1;8(6):e2856–. doi: 10.1038/cddis.2016.457 (PMC5520875; doi:10.1038/cddis.2016.457)
Supplement: Supplementary Figure Legends [file cddis2016457x2.doc]

**Histone deacetylase 3 overexpression in human cholangiocarcinoma and promotion of cell growth via apoptosis inhibition**

Yuyao Yin^1 #^, Mingming Zhang^1 #^, Robert G. Dorfman^2^, Yang Li^1^, Zhenguo Zhao^3^, Yida Pan^4^, Qian Zhou^5^, Shan Huang^6^, Shimin Zhao^5^, Yuling Yao^1 *^, Xiaoping Zou^1 *^

1 Department of Gastroenterology, Nanjing Drum Tower Hospital, the Affiliated Hospital of Nanjing University Medical School, Nanjing University, Nanjing, China.

2 Northwestern University Feinberg School of Medicine, Chicago, IL, USA

3 Department of Surgery, The Affiliated Jiangyin Hospital of Southeast University Medical College, Jiangsu, China.

4 Department of Digestive Diseases of Huashan Hospital, Fudan University, Shanghai, China

5 School of Life Sciences, Fudan University, Shanghai, China

6 Department of Pathology, The Second Hospital of Anhui Medical University, Anhui Medical University, Hefei, China

**#** These authors contribute equally to this work

***Correspondence to:**

Add: No.321 Zhongshan Road, Nanjing 210008, P.R.China

Tel: 86-025-83304616

Yuling Yao, **e-mail:** yaoyulingling@126.com

Add: No.321 Zhongshan Road, Nanjing 210008, P.R.China

Tel: 86-025-83304616

Xiaoping Zou, **e-mail:** 13770771661@163.com

**KEYWORDS**

MI192, apoptosis, cholangiocarcinoma, HDAC3, prognosis

**ABSTRACT**

Histone deacetylase 3 (HDAC3) has an oncogenic role in apoptosis and contributes to the proliferation of cancer cells. MI192 is a novel HDAC 3 specific inhibitor that displays antitumor activity in many cancer cell lines. However, the role of HDAC3 and the antitumor activity of its inhibitor MI192 are not known in cholangiocarcinoma (CCA). The present study aims to identify the target of MI192 in CCA as well as evaluate its therapeutic efficacy. CCK8 and colony-formation assays showed that HDAC3 overexpression promotes proliferation in CCA cell lines. HDAC3 knockdown or treatment with MI192 decreased CCA cell growth and increased caspase-dependent apoptosis, while apoptosis was partially rescued by HDAC3 overexpression. We demonstrated that MI192 can inhibit the deacetylation activity of HDAC3 and its downstream targets *in vitro*, and MI192 inhibited xenograft tumor growth *in vivo.* Immunochemistry showed that HDAC3 was upregulated in CCA tissues compared to adjacent normal tissues, and this was correlated with reduced patient survival. Taken together, these results demonstrate for the first time that MI192 targets HDAC3 and induces apoptosis in human cholangiocarcinoma cells. MI192 therefore shows potential as a new drug candidate for CCA therapy.

**INTRODUCTION**

Cholangiocarcinoma (CCA) is a highly malignant adenocarcinoma with increasing mortality in many countries . Most patients are diagnosed at late stages and are not eligible for surgical resection or liver transplantation. As a result, 5-year survival rates of CCA remain at 10% for the past 3 decades . In addition, as resistance to conventional chemotherapy is becoming increasingly commonplace, research aimed at developing new strategies for treating CCA in the clinic, as well as identifying new tumor markers, is urgently needed.

Histone acetylation is typically associated with increased transcription and histone deacetylases (HDACs) are regulatory enzymes that catalyze the removal of acetyl groups from histones. According to their homology, the 11 HDACs can be divided into Classes I, II and IV . Class I HDACs (1, 2, 3 and 8) play an important role in tumorigenesis and may be candidate targets for many cancer treatments . Numerous reports indicate Class I HDACs are overexpressed in many cancers and inhibit specific tumor suppressor genes, resulting in an aberrant epigenetic status compared to adjacent normal cells . Recently, studies showed that high levels of HDAC3 expression and activity played a critical role in cell epigenetic alterations associated with malignancies . However, the role of HDAC3 in CCA has not been elucidated.

HDAC inhibitors show great potential as promising chemotherapeutic agents because acetylation-mediated epigenetic changes are reversible. Indeed, numerous HDAC inhibitors (Vorinostat (Suberoylanilide Hydroxamic Acid; SAHA), AR-42, romidepsin, entinostat and valproic acid) exhibit anti-tumor effects in a variety of tumors both *in vitro* and *in vivo* . Two HDAC inhibitors, SAHA and romidepsin, are U.S. Food and Drug Administration (FDA) approved for the treatment of cutaneous T-cell lymphoma . MI192, a novel HDAC3 selective inhibitor, was found to be beneficial for rheumatoid arthritis (RA) *in vitro* with marginal toxicity . However, the effects of MI192 in CCA have not yet been studied.

Here we confirmed high levels of HDAC3 in CCA tissues, suggesting poor survival in patients with CCA. We also found that high levels of HDAC3 induced proliferation as well as inhibited apoptosis in CCA cell lines. MI192 inhibited cancer cell proliferation through the induction of cell apoptosis primarily through targeting HDAC3. Thus, HDAC3 inhibition caused apoptosis in CCA cells, and selective inhibition of HDAC3 through novel inhibitors may be useful for CCA therapy.

**RESULTS**

**HDAC3 promoted growth in CCA cells**

Recently studies showed that high levels of HDAC3 expression and activity played a critical role in cell epigenetic alterations associated with malignancies . However, the role of HDAC3 in CCA has not been elucidated. We assessed the expression of HDAC3 in CCA cell lines, and found HDAC3 significantly expressed in all three CCA cell lines (Fig 1A). Next, we evaluated the relationship between cell proliferation and HDAC3 expression using two human CCA cell lines (HuCCT1 and RBE). Transfection of cells with siHDAC3 significantly decreased CCA cell proliferation (sFig A, Fig 1C, 1D & 1E), while HDAC3 overexpression increased CCA cell proliferation (Fig 1F, 1G & 1H). Consistent with proliferation studies, transfection of cells with siHDAC3 significantly decreased colony formation, and HDAC3 overexpression significantly increased CCA cell clonogenicity (Fig 1I, 1J & 1K). Collectively, these results indicate that HDAC3 played a key role in promoting cell proliferation.

**The inhibitory effect of MI192 on CCA cell viability**

CCK8 assay results showed that the 50% growth inhibitory concentration (IC50) of MI192 at 48 h was approximately 6 μM in HuCCT1 cells and RBE cells (Fig 2A, 2B & 2C). Although transfection of cells with HDAC3 plasmid significantly increased cell growth, HDAC3 overexpression hardly reversed the inhibitory effect of MI192 on CCA cells (Fig 2D & 2E). Consistent with CCK8 assays, colony formation in CCA cells was significantly decreased after MI192 treatment (Fig 2F & 2G). Collectively, these results reveal that MI192 treatment reduced CCA cell viability.

**MI192 induced CCA cell apoptosis *in vitro***

To further explore the mechanism of MI192 induced cell proliferative inhibition, flow cytometry was employed, and results demonstrated that MI192 increased the relative amount of cell apoptosis (Fig 3A, 3B & 3C). We investigated the effects of HDAC3 on their downstream targets following MI192 treatment, including acetylated α-Histone3, P53 and Bax . MI192-induced caspase substrate (polyADP ribose polymerase (PARP) and caspase 3) cleavage and P53 expression were mimicked by HDAC3 knockdown (Fig 3D & 3E). Altogether, these results indicate that HDAC3 participated in MI192-induced apoptosis in CCA cells and that HDAC3 might be the target of MI192.

**HDAC3 is the direct target of MI192**

To determine whether MI192 inhibited HDAC3 activity, CCA cells were treated with MI192 following transfection with a HA-tagged HDAC3 vector. We found that HDAC3 protein levels were unchanged by MI192 treatment (Fig 4A). Inhibition of HDAC3 activity by MI192 was subsequently assessed by evaluating specific acetylation of histone H3, the downstream target of HDAC3 . We found that MI192 increased specific histone H3 acetylation, and HDAC3 overexpression reversed this effect (Fig 4D, 4A & 4B).

Because of the high level of homology between the class I HDACs, HDAC 2 shares 52% identity with HDAC3 and MI192 possibly has a weak inhibitory effect on HDAC2 . We then attempted to determine whether HDAC3 was responsible for MI192-induced apoptosis. To elucidate the direct target of MI192, we used an *in vitro* deacetylation system (Fig 4C). MI192 treatment inhibited HDAC3 deacetylation activity, but only had a marginal inhibitory effect on HDAC2 (Fig 4D). We investigated the effects of HDACs 1, 2 and 3 on apoptosis related targets and found that only HDAC3 could rescue the apoptosis signal in CCA cell lines (Fig 4E, 4F & 4G). This data suggests that MI192 inhibited HDAC3 activity.

**Effects of MI192 on tumor xenografts**

We employed a CCA cell tumor xenograft model to evaluate the *in vivo* anti-cancer and HDAC3 inhibitory activity of MI192, and found that MI192 administration significantly inhibited tumor growth (Fig 5A & 5B). The body weights of treated mice were used as indicators of health . MI192 treatment did not affect mouse body weight, suggesting that the mice did not experience evident toxicity *in vivo* (Fig 5C). Furthermore, histological sections of xenograft samples were stained with TUNEL, c-Caspase 3 and Ki-67, markers of cell apoptosis and proliferation, respectively . Consistent with the *in vitro* results, MI192 administration increased TUNEL and c-Caspase 3 staining and reduced Ki-67 staining in xenograft tissues, confirming the anti-tumor effect of MI192 (sFig E, Fig 5D & 5E).

Though other Class I HDACs are found primarily in the nucleus, HDAC3 can shuttle in and out of the nucleus as its catalytic domain is positioned much closer to the C-terminus than other Class I HDACs . We found that HDAC3 mainly localized to the nucleus, but was also observed in the membrane. MI192 treatment did not significantly change the location and protein level of HDAC3 in CCA cell xenograft samples (Fig 5F). Consistent with previous results, we also found that xenografts from HDAC3 knockdown cells were significantly smaller than their counterparts (sFig C & D, Fig 5G & 5H). Altogether, this data demonstrates the apoptosis-inducing and proliferation-inhibiting activity of MI192 *in vivo*.

**HDAC3 expression was increased in CCA tissues and associated with reduced patient survival**

Class I HDACs (especially 1, 2 and 3) play an important role in tumorigenesis and numerous reports indicate that Class I HDACs are overexpressed in many cancers, resulting in an aberrant epigenetic status compared to adjacent normal cells . We queried the tissue microarrays from SHANGHAI OUTDO BIOTECH, which contains clinically annotated data from 127 cholangiocarcinoma samples. When we assessed the expression of HDAC3 on 9 pairs of CCA tissues, we found HDAC3 significantly promoted in tumor tissues compared to adjacent tissues (Fig 6A & 6B). We evaluated the tissue microarrays, which contain clinically annotated genomic data from CCA samples, and found that HDAC3 protein was overexpressed in 47/127 CCA cases (37%), and was associated with tumor size (Table 1). Employing the 33 follow-up cases, we found that high HDAC3 protein in CCA reduced patient survival (P＜0.001, log-rank test) (Fig 6C).

**DISCUSSION**

Numerous reports indicate that HDACs are overexpressed in many cancers and inhibit specific tumor suppressor genes, thereby resulting in aberrant epigenetics in cancer cells . Among them, Class I HDACs play important roles in tumorigenesis. This makes Class I HDACs promising targets for anti-tumor therapeutics . Since modifications in HDAC8 expression did not affect cancer cell proliferation and expression of Class I HDACs in CCA has not yet been studied, we set out to determine the expression of Class I HDACs (especially HDACs 1, 2 and 3) in CCA tissue.

Employing immunohistochemistry, we found that the expression of HDAC3 was differentially expressed and correlated with clinicopathological factors in CCA. We also screened for the effects of HDAC3 on CCA cell proliferation and confirmed that HDAC3 enhances cell proliferation as well as inhibits apoptosis, indicating that HDAC3 could be a potential target of the chemotherapeutic HDAC3 inhibitor.

HDAC 1 and HDAC 2 share 82% identity with each other, as well as share 53% and 52% identity with HDAC3, respectively . Due to the high level of homology between the class I HDACs, it is easy to understand why a HDAC3 selective inhibitor would be difficult to identify. Though MI192, a new class of inhibitor, can show higher selectivity for HDAC3 over HDACs 1 and 2 , its inhibitory effects on other HDACs besides HDAC3 could not be ignored. Therefore, we evaluated the inhibitory effect of MI192 on HDACs 2 and 3 by employing mass spectrometry, and confirmed that MI192 could only significantly inhibit HDAC3 *in vitro*. At the molecular level, HDAC3 overexpression not only partially reversed cell apoptosis, but also reversed apoptosis-related proteins, while HDAC1 and HDAC2 did not show a similar effect. Consistent with *in vitro* data, MI192 significantly inhibited the *in vivo* activity of HDAC3 and induced apoptosis in Hucct1 xenograft tissues. This data suggests that MI192 induces CCA cell apoptosis by inhibiting the activity of HDAC3.

As the catalytic domain of HDAC3 is positioned much closer to the C-terminus than other Class I HDACs, the structure of HDAC3 is distinct from other Class I HDACs . This may explain why HDAC3 protein can shuttle in and out of the nucleus, whereas other Class I HDACs are found primarily in the nucleus . Studies have shown that phosphorylation of a specific serine residue in the HDAC3 protein is regulated by c-Src, kinase CK2, and phosphatase PP4, and that phosphorylation contributes to HDAC3 activity as well as relocation . To elucidate the impact of MI192 on HDAC3, we found that MI192 treatment did not significantly change the cellular location or protein level of HDAC3 in CCA cells and xenograft samples, indicating that MI192 inhibits the deacetylation activity of HDAC3 as opposed to its expression and phosphorylation.

Acetylation increases p53 protein stability, and upon acetylation of p53 at K120, p53 preferentially activates the expression of proapoptotic genes BAX, PUMA, DR5 and NOXA . We evaluated the role of P53 in HDAC3-related apoptosis and found that both HDAC3 knockdown and MI192 treatment significantly increased protein levels of P53 and activated the expression of the downstream proapoptotic gene BAX. HDAC3 overexpression not only rescued cell apoptosis, but also reversed the upregulation of p53 and BAX in CCA cells, indicating that MI192 promotes CCA cell apoptosis partially by increasing HDAC3 acetylation of p53.

In conclusion, the present work found that HDAC3 is a key regulatory factor for cancer proliferation and apoptosis, and is associated with poor prognosis in CCA patients. MI192, as a HDAC inhibitor, represents a novel treatment approach for CCA, and isoform-selective HDAC3 inhibition may improve therapeutic margins of safety. Further characterization of HDAC inhibitors is needed to better establish their role in the management of CCA.

**MATERIALS AND METHODS**

**Ethics, consent and permissions**

All experiments utilizing animal and human samples were approved by the Ethical Com-mittee of Medical Research, Nanjing Drum Tower Hospital, Affiliated Hospital of Nanjing University Medical School.

**Cell culture and reagents**

Three human cholangiocarcinoma (CCA) cell lines were used: HuCCT1, Hccc9810 and RBE. HuCCT1 and Hccc9810 were obtained from the Japanese Collection of Research Bioresources (JCRB) (Tokyo, Japan). RBE was obtained from the Institute of Biochemistry and Cell Biology, Shanghai Institutes for Biological Sciences, Chinese Academy of Sciences (Shanghai, China). Cells were maintained in RPMI-1640 (Invitrogen, Carlsbad, CA, USA) containing 10% fetal bovine serum (Invitrogen), penicillin (Invitrogen) (100 U/ml) and streptomycin (Invitrogen) (100 U/ml). MI192 (Sigma, St Louis, MO, USA) was commercially purchased.

**Immunohistochemistry**

Tumor specimens were fixed in 4% formalin and embedded in paraffin. The sections were incubated with TUNEL kit buffer (Gugebio, Wuhan, China), anti-active Caspase-3 (abcam, Cambridge, UK) or anti-Ki67 antibodies (Santa Cruz, Dallas, TX, USA), and subsequently with DAPI (Gugebio) as well as the corresponding secondary antibody (Zsbio, Beijing, China). Sections were treated with immunoperoxidase using the DAB kit (Zsbio) and then scored . The tissue microarray slides were obtained from OUTDO BIOTECH (Shanghai, China). Staining intensity was graded as follows: absent staining = 0, weak = 1, moderate = 2, and strong = 3. The percentage of staining was graded as follows: 0 (no positive cells), 1 (<25% positive cells), 2 (25% - 50% positive cells), 3 (50% - 75% positive cells), and 4 (>75% positive cells). The score for each tissue was calculated by multiplying, and the range of this calculation was therefore 0 – 12 .

**Cell transfection**

Cells were transfected using Lipofectamine 3000 (Invitrogen) according to the manufacturer’s protocol. The HDAC3 siRNAs were commercially purchased from RiboBio (Guangzhou, China), siRNA-HDAC3-1: CCATGACAATGACAAGGAA, siRNA-HDAC3-2: GCATTGATGACCAGAGTTA, siRNA-HDAC3-3: GAATATGTCAAGAGCTTCA. HDAC3 shRNA (h) Lentiviral Particles were commercially purchased (Santa Cruz). The control vector, HDAC1 - 3 expression vectors were kindly provided by the Zhao lab of Fudan University (Shanghai, China).

**Western blotting analysis**

Cells were lysed with 0.5% NP40 lysis buffer and proteins were blotted following standard protocol. Signals were probed using the chemiluminescence ECL plus reagent (Thermo, Grand Island, NY, USA), as well as detected using the chemiluminescence HRP substrate (Millipore, Billerica, MA, USA) and Tanon 5200Multi scanner (Shanghai, China). Primary antibodies were as follows: HDAC1 (abcam), HDAC2 (abcam), HDAC3 (abcam), cleaved caspase-3 (CST, Danvers, MA, USA), cleaved PARP (CST), PARP (CST), GAPDH (Bioworld, St. Louis Park, MN, USA), K9 acetyl-histone H3 (CST), Bax (CST)，P53 (Santa Cruz), PUMA (CST), HA (CST).

**Cell viability and clonogenic assay**

Cells viability was determined using the CCK-8 colorimetric assay in 96-well plates (2×10^3^ cells/well) (Dijindo, Minato-ku, Tokyo, Japan). The absorbance at 450 nm was recorded using a micro-plate reader. For the clonogenic assay, cells were seeded into 6-well plates (5×10^2^ cells/well) and cultured for 10 days. Colonies were fixed with 4% paraformaldehyde, stained with crystal violet, and then counted.

**Apoptosis assay**

Cell apoptosis was measured by flow cytometry using the AnnexinV-FITC/PI Apoptosis Detection Kit (BD, Franklin Lakes, NJ, USA) following the manufacturer's instructions.

**HDAC deacetylation assay**

Cells were lysed in NP-40 buffer containing 50 mM Tris-HCl (pH 7.5) (Sigma, St Louis, MO, USA), 150 mM NaCl (Sangon, Shanghai, China), 0.5% Nonidet P-40 (Sigma), 1 μg/ml aprotinin (Sigma), 1 μg/ml leupeptin (Sigma), 1 μg/ml pepstatin (Sigma), 1 mM Na_3_VO_4_ (Sigma) and 1 mM PMSF (Sigma). For immunoprecipitation, 500 μl of cell lysate was incubated with HA antibody (provided by the Zhao lab of Fudan University) for three hours at 4˚C with rotation. Then, 30 μl Protein A Agarose (Millipore) was added for 12 hours at 4˚C with rotation, and the beads were washed three times with lysis buffer before proteins were dissolved in loading buffer. Deacetylation assays were carried out in the presence of 5 μg enzyme and 0.3 μg peptide in 30 μl reaction buffer (30 mM HEPES (Sigma), 0.6 mM MgCl_2_ (Sangon), 1 mM DTT (Sigma), 1 mM NAD^+^ (Sigma), 10 mM PMSF (Sigma)). The deacetylation reaction was incubated for 3 - 5 hours at 37˚C before the mixture was desalted by passing it through a C18 ZipTip (Millipore). The desalted samples were analyzed using a MALDI-TOF/TOF mass spectrometer (Applied Biosystems, Grand Island, NY, USA). The acetylated peptide used in the assay was NLASVEELK^Ac^EIDVEVRK (Glssale, Shanghai, China).

**Cholangiocarcinoma cancer xenograft model**

Nude mice were purchased from the Department of Laboratory Animal Science, Nanjing Drum Tower Hospital. HuCCT1 cells (5×10^6^) in FBS-free RPMI-1640 were subcutaneously injected into the flanks of mice. HDAC3 knockdown cells and control counterparts were injected at the left and right sides of the same mice. Once xenograft tumors were palpable, mice were treated with MI192 at a dose of 25 mg/kg bodyweight in 200 μl volume via intraperitoneal injection twice a week for three weeks. Tumor volume was calculated using the formula, length (L) x width (W) x height (H) x 0.5236. The Animal Welfare Committee of Nanjing Drum Tower Hospital approved all procedures involving animals.

**Statistics**

Data was expressed as mean ± standard error of the mean (SE). The data was analyzed through one-way ANOVAs followed by post hoc Duncan tests (SPSS 17.0). P<0.05 was considered significant.

**ACKNOWLEDGMENTS**

We thank the Zhao lab for offering their help.

**GRANT SUPPORT**

This work was supported by grants from the National Natural Science Foundation of China (No. 81602076, No. 81400306, No. 81401977 and No. 81401974), the Natural Science Foundation from the Department of Science & Technology of Jiangsu Province (BK20160113), General Project of Nanjing Municipal Bureau of Health (No. YKK12053), Outstanding Youth Project of Nanjing City (No. JQX14005) and the Fundamental Research Funds for the Central Universities (No. 021414380244).

**CONFLICTS OF INTEREST**

The authors declare no conflicts of interest.

**REFERENCES**

1. Pinter M, Hucke F, Zielonke N, Waldhor T, Trauner M, Peck-Radosavljevic M and Sieghart W. Incidence and mortality trends for biliary tract cancers in Austria. Liver Int. 2014; 34(7):1102-1108.

2. von Hahn T, Ciesek S, Wegener G, Plentz RR, Weismuller TJ, Wedemeyer H, Manns MP, Greten TF and Malek NP. Epidemiological trends in incidence and mortality of hepatobiliary cancers in Germany. Scand J Gastroenterol. 2011; 46(9):1092-1098.

3. Plentz RR and Malek NP. Clinical presentation, risk factors and staging systems of cholangiocarcinoma. Best Pract Res Clin Gastroenterol. 2015; 29(2):245-252.

4. Rizvi S and Gores GJ. Pathogenesis, diagnosis, and management of cholangiocarcinoma. Gastroenterology. 2013; 145(6):1215-1229.

5. Lakshmaiah KC, Jacob LA, Aparna S, Lokanatha D and Saldanha SC. Epigenetic therapy of cancer with histone deacetylase inhibitors. J Cancer Res Ther. 2014; 10(3):469-478.

6. Mottet D, Pirotte S, Lamour V, Hagedorn M, Javerzat S, Bikfalvi A, Bellahcene A, Verdin E and Castronovo V. HDAC4 represses p21(WAF1/Cip1) expression in human cancer cells through a Sp1-dependent, p53-independent mechanism. Oncogene. 2009; 28(2):243-256.

7. Zhu C, Chen Q, Xie Z, Ai J, Tong L, Ding J and Geng M. The role of histone deacetylase 7 (HDAC7) in cancer cell proliferation: regulation on c-Myc. J Mol Med (Berl). 2011; 89(3):279-289.

8. Vigushin DM and Coombes RC. Histone deacetylase inhibitors in cancer treatment. Anticancer Drugs. 2002; 13(1):1-13.

9. Song SH, Han SW and Bang YJ. Epigenetic-based therapies in cancer: progress to date. Drugs. 2011; 71(18):2391-2403.

10. Arts J, King P, Marien A, Floren W, Belien A, Janssen L, Pilatte I, Roux B, Decrane L, Gilissen R, Hickson I, Vreys V, Cox E, Bol K, Talloen W, Goris I, et al. JNJ-26481585, a novel "second-generation" oral histone deacetylase inhibitor, shows broad-spectrum preclinical antitumoral activity. Clin Cancer Res. 2009; 15(22):6841-6851.

11. Witt O, Deubzer HE, Milde T and Oehme I. HDAC family: What are the cancer relevant targets? Cancer Lett. 2009; 277(1):8-21.

12. Mariadason JM. HDACs and HDAC inhibitors in colon cancer. Epigenetics. 2008; 3(1):28-37.

13. West AC and Johnstone RW. New and emerging HDAC inhibitors for cancer treatment. J Clin Invest. 2014; 124(1):30-39.

14. Barneda-Zahonero B and Parra M. Histone deacetylases and cancer. Mol Oncol. 2012; 6(6):579-589.

15. Stimson L, Wood V, Khan O, Fotheringham S and La Thangue NB. HDAC inhibitor-based therapies and haematological malignancy. Ann Oncol. 2009; 20(8):1293-1302.

16. Piekarz RL, Frye R, Turner M, Wright JJ, Allen SL, Kirschbaum MH, Zain J, Prince HM, Leonard JP, Geskin LJ, Reeder C, Joske D, Figg WD, Gardner ER, Steinberg SM, Jaffe ES, et al. Phase II multi-institutional trial of the histone deacetylase inhibitor romidepsin as monotherapy for patients with cutaneous T-cell lymphoma. J Clin Oncol. 2009; 27(32):5410-5417.

17. Olsen EA, Kim YH, Kuzel TM, Pacheco TR, Foss FM, Parker S, Frankel SR, Chen C, Ricker JL, Arduino JM and Duvic M. Phase IIb multicenter trial of vorinostat in patients with persistent, progressive, or treatment refractory cutaneous T-cell lymphoma. J Clin Oncol. 2007; 25(21):3109-3115.

18. Zhang M, Pan Y, Dorfman RG, Chen Z, Liu F, Zhou Q, Huang S, Zhang J, Yang D and Liu J. AR-42 induces apoptosis in human hepatocellular carcinoma cells via HDAC5 inhibition. Oncotarget. 2016.

19. Boumber Y and Issa JP. Epigenetics in cancer: what's the future? Oncology (Williston Park). 2011; 25(3):220-226, 228.

20. Gillespie J, Savic S, Wong C, Hempshall A, Inman M, Emery P, Grigg R and McDermott MF. Histone deacetylases are dysregulated in rheumatoid arthritis and a novel histone deacetylase 3-selective inhibitor reduces interleukin-6 production by peripheral blood mononuclear cells from rheumatoid arthritis patients. Arthritis Rheum. 2012; 64(2):418-422.

21. Jiao F, Hu H, Yuan C, Jin Z, Guo Z and Wang L. Histone deacetylase 3 promotes pancreatic cancer cell proliferation, invasion and increases drug-resistance through histone modification of P27, P53 and Bax. Int J Oncol. 2014; 45(4):1523-1530.

22. Hagelkruys A, Sawicka A, Rennmayr M and Seiser C. The biology of HDAC in cancer: the nuclear and epigenetic components. Handb Exp Pharmacol. 2011; 206:13-37.

23. Yang WM, Tsai SC, Wen YD, Fejer G and Seto E. Functional domains of histone deacetylase-3. J Biol Chem. 2002; 277(11):9447-9454.

24. Yang WM, Yao YL, Sun JM, Davie JR and Seto E. Isolation and characterization of cDNAs corresponding to an additional member of the human histone deacetylase gene family. J Biol Chem. 1997; 272(44):28001-28007.

25. Boissinot M, Inman M, Hempshall A, James SR, Gill JH, Selby P, Bowen DT, Grigg R and Cockerill PN. Induction of differentiation and apoptosis in leukaemic cell lines by the novel benzamide family histone deacetylase 2 and 3 inhibitor MI-192. Leuk Res. 2012; 36(10):1304-1310.

26. Wang LT, Liou JP, Li YH, Liu YM, Pan SL and Teng CM. A novel class I HDAC inhibitor, MPT0G030, induces cell apoptosis and differentiation in human colorectal cancer cells via HDAC1/PKCdelta and E-cadherin. Oncotarget. 2014; 5(14):5651-5662.

27. Ler SY, Leung CH, Khin LW, Lu GD, Salto-Tellez M, Hartman M, Iau PT, Yap CT and Hooi SC. HDAC1 and HDAC2 independently predict mortality in hepatocellular carcinoma by a competing risk regression model in a Southeast Asian population. Oncol Rep. 2015; 34(5):2238-2250.

28. Wu LM, Yang Z, Zhou L, Zhang F, Xie HY, Feng XW, Wu JA and Zheng SS. Identification of Histone Deacetylase 3 as a Biomarker for Tumor Recurrence Following Liver Transplantation in HBV-Associated Hepatocellular Carcinoma. PLoS One. 2010; 5(12).

29. Malvaez M, McQuown SC, Rogge GA, Astarabadi M, Jacques V, Carreiro S, Rusche JR and Wood MA. HDAC3-selective inhibitor enhances extinction of cocaine-seeking behavior in a persistent manner. Proc Natl Acad Sci U S A. 2013; 110(7):2647-2652.

30. Zhang X, Ozawa Y, Lee H, Wen YD, Tan TH, Wadzinski BE and Seto E. Histone deacetylase 3 (HDAC3) activity is regulated by interaction with protein serine/threonine phosphatase 4. Genes Dev. 2005; 19(7):827-839.

31. Longworth MS and Laimins LA. Histone deacetylase 3 localizes to the plasma membrane and is a substrate of Src. Oncogene. 2006; 25(32):4495-4500.

32. Kim SS and Benchimol S. HDAC5--a critical player in the p53 acetylation network. Mol Cell. 2013; 52(3):289-290.

33. Brown RS and Wahl RL. Overexpression of Glut-1 Glucose-Transporter in Human Breast-Cancer - an Immunohistochemical Study. Cancer. 1993; 72(10):2979-2985.

34. Xiao Y, Wang J, Qin Y, Xuan Y, Jia Y, Hu W, Yu W, Dai M, Li Z, Yi C, Zhao S, Li M, Du S, Cheng W, Xiao X, Chen Y, et al. Ku80 cooperates with CBP to promote COX-2 expression and tumor growth. Oncotarget. 2015; 6(10):8046-8061.

FIGURE LEGENDS

**Figure 1: HDAC3 promoted growth in CCA cells.**

(A) HDAC3 protein levels were detected by western blot. (B and C) HuCCT1 and RBE cell proliferation was analyzed via CCK8 assay following siRNA transfection. (D) Transfection efficiency was confirmed by western blotting. (E and F) HuCCT1 and RBE cell proliferation was analyzed via CCK8 assay following transfection with HDAC3 overexpression vector. (G) Transfection efficiency was confirmed by western blotting. (H) Cells were transfected with HDAC3 overexpression vector or siRNAs, then colonies were stained with crystal violet and photographed. Scale bars, 1 cm. (I and J) Stained colonies were quantified. Data represent the mean ± SEM, n≥3. *p<0.05, **p<0.01.

**Figure 2: The inhibitory effect of MI192 on CCA cell viability.** (A and B) Cells were treated with MI192 and quantified via CCK-8 assay. (C) Cells were treated with MI192 and morphological changes were observed. The magnification is x200. Scale bars, 100 μm. (D and E) Cells were transfected with HDAC3 overexpression vector, then treated with MI192 and quantified via CCK-8 assay. (F and G) Cells were treated with MI192. Colonies were stained with crystal violet (left) and quantified (right). Scale bars, 1 cm.

**Figure 3: MI192 induced CCA cell apoptosis *in vitro*.**

(A) Cells were treated with MI192, and cell apoptosis was analyzed via flow cytometry. (B and C) Cell apoptosis was quantified. (D and E) Cells were collected and subjected to western blot after MI192 treatment (left) and siRNA transfection (right).

**Figure 4: HDAC3 is the direct target of MI192.**

(A and B) HDAC3-overexpressing cells were treated with MI192 and subjected to western blot. (C) Schematic diagram of the *in vitro* deacetylation assay with HDAC3 (top). The immunoprecipitated protein corresponding to HDACs-HA was subjected to western blot (bottom). (D) The HDACs protein was incubated with acetylated peptides with or without MI192, and the rate of deacetylation was determined using Mass Spectrometry (MS). (E) HDAC1-overexpressing cells and their counterparts were subjected to western blot. (F) HDAC2-overexpressing cells and their counterparts were subjected to western blot. (G) HDAC3-overexpressing cells and their counterparts were subjected to western blot. Data represent the Mean ± SEM, n≥3. *p<0.05, **p<0.01, NS not significant.

**Figure 5: Effects of MI192 on tumor xenografts.**

(A) Systemic delivery of MI192 suppresses CCA cell xenograft tumor growth in nude mice. Tumors were photographed after all animals were sacrificed. Scale bars, 1 cm. (B) The xenograft tumor sizes. (C) The body weights of tumor-burdened mice. (D) Xenograft samples were stained with Ki-67 (left) and staining was quantified (right). The magnification is x200. Scale bars, 100 μm. (E) Xenograft samples were stained with TUNEL (left) and staining was quantified (right). The magnification is x200. Scale bars, 100 μm. (F) Xenograft samples were stained with HDAC3 (left) and staining was quantified (right). The magnification is x200. Scale bars, 100 μm. (G) HDAC3 knockdown HuCCT1 cells and control counterparts were injected at the left and right sides of the same mice. Tumors were photographed after all animals were sacrificed. Scale bars, 1 cm. (H) Xenograft tumor sizes. Data represent the mean ± SEM, n≥3. *p<0.05, **p<0.01, NS not significant.

**Figure 6: HDAC3 expression was increased in CCA tissues.**

(A and B) HDAC3 protein levels in tumor and adjacent normal tissues from 9 CCA patients were detected (top) and quantified (bottom). The magnification is x200. Scale bars, 100 μm. (C) 5-year survival was reduced for CCA patients with elevated HDAC3 protein expression. Data represent the mean ± SEM, n≥3. *p<0.05, **p<0.01.

**Supplementary Figure:**

(A) The knockdown efficiency of HDAC3 siRNA was detected by western blot. (B) HDAC3 mRNA levels were dectected in CCA cells by q-PCR. (C) HDAC3 knockdown efficiency in HuCCT1 cells was detected by western blot. (D) HDAC3 levels in xenotransplanted tumors. (E) Cleaved caspase 3 levels in xenograft tumors. Data represent the mean ± SEM, n≥3. *p<0.05, **p<0.01.
